# Hazard Management Dealt by Safety Professionals in Colleges: The Impact of Individual Factors

**DOI:** 10.3390/ijerph13121201

**Published:** 2016-12-03

**Authors:** Tsung-Chih Wu, Chi-Hsiang Chen, Nai-Wen Yi, Pei-Chen Lu, Shan-Chi Yu, Chien-Peng Wang

**Affiliations:** 1 Department of Industrial Education and Technology, National Changhua University of Education, Changhua 50074, Taiwan; ccicjs2011@gmail.com (C.-H.C.); a149958@yahoo.com.tw (N.-W.Y.); w661382@yahoo.com.tw (P.-C.L.); shanchi137@gmail.com (S.-C.Y.); 2 Department of Industrial Safety, Ju-Kao Engineering Co., Ltd., Taichung 43541, Taiwan; piggy1234567@hotmail.com

**Keywords:** safety professional, hazard management, individual factors

## Abstract

Identifying, evaluating, and controlling workplace hazards are important functions of safety professionals (SPs). The purpose of this study was to investigate the content and frequency of hazard management dealt by safety professionals in colleges. The authors also explored the effects of organizational factors/individual factors on SPs’ perception of frequency of hazard management. The researchers conducted survey research to achieve the objective of this study. The researchers mailed questionnaires to 200 SPs in colleges after simple random sampling, then received a total of 144 valid responses (response rate = 72%). Exploratory factor analysis indicated that the hazard management scale (HMS) extracted five factors, including physical hazards, biological hazards, social and psychological hazards, ergonomic hazards, and chemical hazards. Moreover, the top 10 hazards that the survey results identified that safety professionals were most likely to deal with (in order of most to least frequent) were: organic solvents, illumination, other chemicals, machinery and equipment, fire and explosion, electricity, noise, specific chemicals, human error, and lifting/carrying. Finally, the results of one-way multivariate analysis of variance (MANOVA) indicated there were four individual factors that impacted the perceived frequency of hazard management which were of statistical and practical significance: job tenure in the college of employment, type of certification, gender, and overall job tenure. SPs within colleges and industries can now discuss plans revolving around these five areas instead of having to deal with all of the separate hazards.

## 1. Introduction

### 1.1. Background

Education is a valuable means for learning knowledge, skills, and attitudes. A system of schooling has continually developed and matured over time, gradually forming standardized school classifications. Among the numerous school classifications, universities are defined as institutions of higher education that provide comprehensive education and research opportunities and are authorized to award academic degrees. According to the University Act of Taiwan [1], universities are senior educational organizations established according to the Act that grant academic degrees above the bachelor’s degree (inclusive), encourage academic research, cultivate talent, enhance culture, serve society, and accelerate the development of the country.

Because of rapid social change and the increasing demand for higher education in recent years, the number of universities in Taiwan has increased exponentially, and universities have changed from elitist to mass-oriented education models [2]. Universities not only utilize classrooms, media, the Internet, and other hardware facilities to provide students with relevant knowledge and skills, but also establish laboratories, test rooms, internship sites, and experimentation sites to reinforce students’ practical skills and application of theory [3]. However, such spaces, particularly experimentation sites, present potential risks and hazards, regardless of how these spaces are used (e.g., research or teaching practice) [4].

To enhance safety and health management in college experimentation sites, college laboratories, test rooms, internship workshops, and experimentation sites have been included in the applicable scope of the *Occupational Safety and Health Act* [5]. In addition, the Ministry of Education formulated the *Environment and Safety Management in Universities and Performance Evaluation Work Plan* to inspect and evaluate the environment and safety performance of universities in an attempt to improve environmental protection and resource utilization in universities, enhance the performance of safety and health management facilities and personnel, and encourage universities to establish a comprehensive management system and self-check mechanism, thereby fulfilling college campus environment and safety management tasks, maintaining the college environment, and achieving safety management [6].

Despite the efforts of the government and relevant authorities in promoting college campus safety and health management, major hazardous events at colleges have occasionally been reported in recent years. Taiwan Higher Education Union noted that 32 accidents occurred in Taiwanese college laboratories that caused deaths and injuries to employees during the period 1997–2014 [7]. Not only should the government push relevant regulations to improve safety and health management in universities and reduce disasters and losses, but safety professionals (SPs) with extensive knowledge in safety and health management should also be commissioned to conduct onsite inspections. Subsequently, the content of college SP hazard management must be examined to identify the potential hazards that SPs manage, confirm the various orders of frequency of managing hazards, and clarify the factors that may influence the frequencies of SP hazard management, thereby facilitating the effective execution of hazard management tasks.

### 1.2. Conceptualizing and Operationalizing Hazard

The *Dictionary of Terms Used in the Safety Profession* published by the American Society of Safety Engineers [8] defined hazard as the change in a single situation or a series of situations that results in potential harm, illness, or property damage or the potential of a task, situation, environment, or innate quality to cause unfavorable or harmful outcomes. Brauer defined hazard as the changes in environmental-setting conditions that result in harm, illness, or property damage or the extant or potential characteristics of an event, situation, or environment that may produce unfavorable or harmful outcomes [9]. Therefore, the present study defined hazard as the harmful operations, situations, or environment within a workplace that influence safety and health.

The National Safety Council classified hazards into four types: chemical hazards, physical hazards, biological hazards, and ergonomic hazards [10]. Lu supported these hazard classifications and further asserted that hazards relating to industrial hygiene also belong to these classifications [11]. In addition to the four common hazard classifications, sociological and psychological hazards have gradually gained social awareness [12]. Ju indicated that the negative influences that employee psychology has on work performance have become increasingly severe in recent years [13]. Subsequently, Council of Labor Affairs, Executive Yuan, Taiwan announced the *Technical Guidelines for Hazard Identification and Risk Assessment*, which classified hazards into five types: physical, chemical, biological, ergonomic, and psychological hazards [14]. Therefore, the present study classified hazards into five types: physical, chemical, biological, ergonomic, and sociological and psychological hazards.

### 1.3. Hazards Dealt by Safety Professionals

Safety Institute of Australia Ltd. (Victoria, Australia) [15] argued “*Organizations may be considered a system which may contain hazards which must be under control to minimize risk. This can be achieved by understanding models causation for safety and for health which will result in improvement in the safety and health of people at work*”. Khanzode, Maiti, and Ray comprehensively reviewed the concepts of occupational injury and accident causation, and then proposed three intervention strategies including engineering interventions, behavioral interventions, and enforcement interventions [16]. With regard to enforcement interventions in preventing injuries/accidents, there could be two important approaches: governmental labor inspection and industrial self-inspection. The SPs play an important role in the self-inspection within industries.

The Board of Certified Safety Professionals [17] defined a safety professional as “*a person engaged in the prevention of accidents, incidents, and events that harm people, property, or the environment*”. Industry SPs in Taiwan are defined as personnel engaged in planning and monitoring safety and health in the workplace to ensure the safety and health of workers, including labor safety and health directors, labor safety specialists, labor health specialists, and labor safety and health managers [18].

The American National Standard Institute (ANSI) normalized the scope and functions of the professional safety position as such: (1) anticipate, identify, and evaluate hazardous conditions and practices; (2) develop hazard control designs, methods, procedures, and programs; (3) implement, administer, and advise others on hazard controls and hazard control programs; (4) measure, audit, and evaluate the effectiveness of hazard controls and hazard control programs [19]. The American Society of Safety Engineers Foundation (ASSEF) and the Board of Certified Safety Professionals (BCSP) [20] indicated that most SPs undertake the following functions: hazard recognition, inspections/audits, fire protection, regulatory compliance, health hazard control, ergonomics, hazardous material management, environmental protection, training, accident and incident investigations, advising management, record keeping, evaluating, emergency response, managing safety programs, product safety, and security.

In related empirical studies, many authors [21,22,23,24,25,26,27] indicated that hazard management is one of SPs’ safety functions. Specifically, Wu and Feng suggested that the top three hazard types handled by industry SPs are chemical, physical, and ergonomic hazards [28]. However, universities with numerous laboratories were very rarely audited by SPs [29,30].

### 1.4. Objectives

Using an exploratory factor-analysis approach, Wu and Feng classified hazards in science and industrial parks that SPs are required to address into four types: ergonomic, physical, biological, and chemical hazards [28]. However, potential hazard classifications may differ for universities because of their functional and structural differences with industrial organizations. Therefore, the objective of the present study was to examine the content and frequency of hazard management by college SPs and to determine factors that influence the performance of hazard management. The primary objective of the present study was divided into the following goals: (1) develop a hazard management scale (HMS) for college SPs; (2) examine the orders of frequency of SP hazard management; and (3) examine the influences that different organizational or individual factors have on the hazard management of college SPs.

## 2. Methods

### 2.1. Population and Samples

College SPs are responsible for executing safety and health tasks on a college campus. Based on the research objective, the present study selected the SP body responsible for college occupational safety and health (OSH) management in Taiwan as the target population. Because a national college SP communications network had not yet been established at the time of research (December 2009), the study collected the SP information of individual colleges from the Internet and compiled a list of 256 SPs from 131 universities. This list was adopted as the accessible population for research. The population comprised 102 SPs from colleges in Northern Taiwan (39.8%), 68 from colleges in Central Taiwan (26.6%), and 86 from colleges in Southern Taiwan (33.6%). Among these SPs, 93 served in public colleges (36.3%) and 163 served in private colleges (63.7%).

“*Although there are many problems to overcome and many pitfalls lurking for the uneducated researcher, a mail survey is a very appropriate way of gathering data and can produce high quality information*” [31]. A simple random sampling method was employed to select 200 SPs from the population, and a questionnaire was mailed to the selected SPs. Among the 200 recipients, 79 served in colleges in Northern Taiwan (39.5%), 53 served in colleges in Central Taiwan (26.5%), and 68 served in colleges in Southern Taiwan (34%). Additionally, 71 of the 200 recipients served in public colleges (35.5%), and the remaining recipients served in private colleges (64.5%). The questionnaire was sent as a package to the recipients in mid-December of 2009. A sealed envelope with the recipient’s name and department was attached to the package. The package comprised a questionnaire, souvenir, and prepaid return envelope.

### 2.2. Instrument

A questionnaire survey was adopted in the present study. The research tool consisted of two parts. The first part was a demographics survey to test organizational and personal factors. The second part was a hazard management scale (HMS) to test the perceptions of college SPs toward frequencies of managing hazards. The HMS developed in the present study was a revised version of the questionnaire for safety professionals proposed by Hale et al. [32]. The HMS consisted of 28 items divided into five subscales: six items of physical hazards, five of chemical hazards, five of biological hazards, seven of ergonomic hazards, and five of sociological and psychological hazards. The recipients were instructed to assume the role of an SP and evaluate their frequency of managing hazards. For the scoring system, the study adopted a 5-point Likert scale ranging from 5 to 1: 5, “weekly”; 4, “monthly”; 3, “quarterly”; 2, “annually”; and 1, “never”. The researcher in this study conducted an onsite observation to identify the potential hazards, and then confirmed the applicability of the instrument.

### 2.3. Data Analysis

Data processing and analysis were performed using SPSS Version 12.0 statistical software (SPSS, Inc., Chicago, IL, USA). Statistical methods included descriptive statistics, exploratory factor analysis (EFA), reliability analysis (RA), and one-way multivariate analysis of variance (MANOVA). Descriptive analysis provided an overview of the various samples, the EFA analyzed various factors and construct validity, the RA tested the internal consistency of the HMS, and the one-way MANOVA examined the differences between organizational and individual factors regarding SP hazard management.

### 2.4. Ethical Statement

All subjects gave their informed consent for inclusion before they participated in the study. The study was conducted in accordance with the Declaration of Helsinki, and the protocol was approved by the Human Experiment and Ethics Committee of Hung Kuang University (Project identification code: 98-B-016).

## 3. Results and Discussion

### 3.1. Sample Response Overview

The questionnaires were distributed during December 2009. After two reminder letters, 144 valid questionnaires were recovered, yielding a recovery rate of 72%. The response rates of round one to round three were 49%, 9%, and 14%, respectively.

To determine whether differences existed in samples collected at different stages, the study first categorized the 144 respondents into a “nonreminder group,” which comprised 98 respondents, and a “reminder group,” which comprised 46 respondents, and then individually performed MANOVA on each group. The results showed no significant difference for respondents’ perceptions concerning the frequency of managing hazards between the nonreminder group and those in the reminder group (Wilks’ Λ = 0.956, *df* = 5, *p* = 0.285). Therefore, the two groups could be combined for subsequent analyses.

Among the 144 respondents, 104 were men (72.2%) and 40 were women (27.8%). The average age of the respondents was 42 years (SD = 8.68). Fifty of the respondents served in public colleges (34.7%) and 94 served in private colleges (65.3%). Regarding location, 45 respondents were from Northern Taiwan (31.3%), 46 were from Central Taiwan (31.9%), and 53 were from Southern Taiwan (36.8%). A chi-square test was conducted to determine whether the properties and geographical locations of the responding sample were homogeneous with the sample population. The results showed no statistically significant difference between the sample population and the properties and geographical locations of the responding sample (properties: χ^2^ = 0.103, *df* = 1, *p* = 0.748; geographical location: χ^2^ = 3.062, *df* = 2, *p* = 0.216), suggesting that the sample distribution of the present study was similar to the sample population.

### 3.2. Exploratory Factor Analysis

#### 3.2.1. Item Analysis

The item analysis results are tabulated in the Appendix (Table A1). The mean values (M) for the 28 items ranged between 1.26 and 2.14, and the standard deviation (SD) ranged between 0.74 and 1.20. The product-moment correlations for the various items and total score of the HMS were between 0.67 and 0.84, indicating that all of the items reached significant and positive correlations (*p* < 0.01). These results further implied that the items were consistent with the overall scale and that each of the items possessed appropriate distinguishing power [33].

#### 3.2.2. Validity Analysis

The construct validity of the HMS was tested using EPA. First, a fitness test was conducted on the factors. The results showed that the Kaiser-Meyer-Olkin (KMO) sampling measure of appropriateness was 0.93. In addition, the Bartlett sphericity test revealed an approximate chi-square value with a significant value (χ^2^ = 4556.83, *p* < 0.001), suggesting that the data employed in the present study were appropriate for factor analysis [34].

After confirming appropriateness, the study commenced factor analysis. A principle component analysis was performed to extract the various factors, and the Varimax method was employed for orthogonal rotation. Hair, Black, Babin, Anderson, and Tatham suggested that items with factor-loading values less than 0.50 (H20, cold or heat) should be omitted [35]. After a secondary EFA, three items presented significantly lower correlations than the other extracted factors: H6 (ionized and nonionized radiation), H7 (dust), and H9 (lead). Thus, the three items were omitted from the present study.

Finally, five factors were obtained from the HMS: physical, biological, ergonomic, sociological and psychological, and chemical hazards, as shown in Table 1. The correlation (factor-loading) values between the various factors and items were between 0.54 and 0.86, suggesting that all of the items achieved practical significance according to Hair et al. [35]. The eigenvalues of the five factors were 4.53, 4.16, 3.72, 3.58, and 3.52 for physical, biological, ergonomic, sociological and psychological, and chemical hazards, respectively. Kaiser suggested that factors with eigenvalues greater than 1 should be retained during factor analysis because such factors demonstrate favorable explanatory power [36]. Thus, all five factors identified in the present study demonstrated favorable explanatory power. In addition, Cattell suggested that the number of factors to retain could be determined using a scree plot, where the sharp decline before a stabilization point indicates the number of factors to retain [37]. The scree plot produced in the factor analysis of the present study showed that all five factors should be retained. The explained variance percentiles of physical, biological, ergonomic, sociological and psychological, and chemical hazards were 18.88%, 17.34%, 15.51%, 14.91%, and 14.66%, respectively. The five factors explained 81.30% of the overall variance of hazard management.

#### 3.2.3. Reliability Analysis

The alpha coefficient proposed by Cronbach was employed to test the internal consistency of the proposed HMS [38]. The results showed that the Cronbach’s α coefficient for the subscales of physical, biological, sociological and psychological, ergonomic, and chemical hazards were 0.94, 0.92, 0.93, 0.93, and 0.92, respectively. The Cronbach’s α coefficient for the entire scale was 0.97. According to Nunnally, a Cronbach’s α coefficient higher than 0.7 represents a favorable internal consistency [39].

### 3.3. Order of Frequency of Managing Hazards

The various frequencies of the hazard management of college SPs are tabulated in Table 2. The table shows that the mean values for various hazards ranged between 1.26 and 2.14. The three most common hazard types handled by SPs were chemical hazards (M = 1.99), physical hazards (M = 1.86), and ergonomic hazards (M = 1.64). Regarding the overall ranking of hazardous items, “organic solvents”, “illumination”, and “other chemical substances” were ranked first, second, and third, respectively. Among the five hazard types, the three most commonly handled hazardous items in each type were “illumination”, “machinery and equipment”, and “fire and explosion” for physical hazards; “bacteria”, “animals”, and “fungi” for biological hazards; “organic solvents”, “other chemical substances”, and “specific chemical substances” for chemical hazards; “human error”, “lifting and carrying”, and “working posture” for ergonomic hazards; and “job stress”, “workplace violence”, and “sexual harassment” for sociological and psychological hazards.

Notably, “organic solvents” and “other chemical substances” in chemical hazards and “illumination” in physical hazards were the most common hazardous problems handled by SPs. These substances are commonly employed in the science, engineering, agriculture, and medical departments of universities, and more attention should be focused on matters concerning these substances. Wu and Feng indicated that the three most common hazard types handled by science park SPs are chemical, physical, and ergonomic hazards [28], which is a similar conclusion to that of the present study. Regarding the ranking of various hazardous items, the most common items handled by science park SPs included organic solvents, machinery and equipment, noise, and illumination. These results are also consistent with those of the present study. Because Wu and Feng primarily selected science park SPs as research subjects, their institutional characteristics differed from those experienced by the participants in the present study, resulting in slight differences in the results [28]. However, “organic solvents” was the most common hazardous item handled by SPs, whether they served in science parks or universities.

### 3.4. One-Way MANOVA

#### 3.4.1. The Central Location and Variation of Hazard Management Perceptions

The mean value (M) for the hazard management perception conditions of college SPs was 1.65 with a standard deviation (SD) of 0.76, with the frequency of execution approximately ranging between “once a year” and “never.” Regarding the various dimensions, the mean values for physical, biological, sociological and psychological, ergonomic, and chemical hazards were 1.86, 1.53, 1.34, 1.64, and 1.99, respectively. These results showed that the college SPs’ perceptions concerning the frequency of managing hazards ranged between “once a year” and “never”, with the frequency of handling chemical hazards being the highest at approximately once a year. The frequency of handling sociological and psychological hazards was the lowest.

In addition, the results of a one-way repeated measures MANOVA showed that the frequencies for managing the five hazard types achieved significant differences (Wilks’ Λ = 0.594, *df* = 4, *p* < 0.001). The results of a pair-wise comparisons test further showed that, in addition to biological and ergonomic hazards (which failed to achieve significant differences), the frequencies of managing the remaining hazard types achieved significant differences with each other (Table 3).

The aforementioned results may be correlated with the competence of college departments and offices. When a chemical, physical, biological, or ergonomic hazard occurs on campus, faculty members may report the incident to the environment safety unit or general services office. Therefore, SPs more frequently handle these types of hazard. However, when a sociological or psychological hazard occurs, such as sexual assault, workplace violence, or job stress, faculty members may seek the assistance of the counseling center or the academic affairs office. Thus, this may account for why SPs handle these types of hazard less frequently.

#### 3.4.2. One-Way MANOVA

A one-way MANOVA was performed to examine the influences that four organizational factors (geological location, size, nature, and presence of an OSH unit) and 10 personal factors (gender, age, years of OSH experience, job tenure in the college of employment, job title, work status, level of education, highest major, type of certification, basis of executing task) have on the frequency of managing hazards. The results showed that “job tenure in the college of employment” and “type of certification” significantly influenced the frequency of managing hazards. “Gender” and “years of OSH experience” failed to significantly influence the frequency of managing hazards but achieved practical significance. The remaining 10 factors failed to significantly influence the frequency of managing hazards.

Statistical significance: Analysis results showed that perceptions concerning the frequency of managing hazards of recipients at various colleges and “years of OSH experience” achieved significant differences (Wilks’ Λ = 0.909, *df* = 5, *p* = 0.025, η^2^ = 0.091). The ANOVA results showed that respondents with 10 or more years of OSH experience demonstrated higher perceptions concerning the frequency of managing physical hazards than those with 9 years or less (Table 4).

Respondents with longer tenure in a single college were more familiar with the various facilitates available and the different operations employed in a college and were possibly more proficient at handling hazards involving electrical and mechanical equipment, fires, and explosions. Therefore, respondents with 10 or more years of tenure at a single college demonstrated higher perceptions concerning the frequency of managing physical hazards than those with 9 years or less. Gyekey indicated that years of service correlated with workplace hazard identification, in which respondents with more years of service demonstrated increased sensitivity to hazardous situations [40]. Therefore, respondents with more years of service may demonstrate greater safety awareness and hazard sensitivity and may be more capable of effectively identifying, confirming, and handling hazards. In this context, respondents with more years of service demonstrated higher perceptions concerning the frequency of managing hazards than those with less years of service.

Moreover, the MANOVA results showed that the “type of certification” of the respondents achieved statistical differences (Wilks’ Λ = 0.788, *df* = 15, *p* = 0.010, η^2^ = 0.076). However, these differences were not significant in the ANOVA results. Consequently, only the practical significance of certification is discussed in the present study.

Practical significance: The results achieving statistical significance suggest that such results possess only statistical meaning. Data should achieve not only statistical significance but also practical significance [34,41] because statistical significance becomes easier to achieve as the size of the sample population increases.

Regarding the criteria for practical significance, Cohen explained that a η^2^ value between 6% and 14% indicates a moderate correlation and a η^2^ value over 14% indicates a strong correlation [42]. For example, the “tenure in the college of employment” item achieved not only statistical significance but also practical significance (Wilks’ Λ = 0.909, *df* = 5, *p* = 0.025, η^2^ = 0.091). In addition, “type of certification” achieved both statistical and practical significance in the MANOVA results but failed to achieve significant difference with any of the hazard types in the ANOVA results. Therefore, only the practical significance of certification is discussed in the present study. “Gender” (Wilks’ Λ = 0.936, *df* = 5, *p* = 0.101, η^2^ = 0.064) and “years of OSH experience” (Wilks’ Λ = 0.935, *df* = 5, *p* = 0.104, η^2^ = 0.065) failed to achieve statistical significance but achieved practical significance.

The different channels through which SPs acquire their certification may influence their hazard identification ability, understanding of regulations, and hazard management methods, consequently affecting their hazard management performance. SPs who can effectively identify hazards and select appropriate management approaches can handle more hazards more efficiently. Chen found differences in the understanding of regulations, understanding of construction site hazards, and frequency of site inspections of SPs with different certifications [43]. Thus, SPs with different types of certification exhibit differing safety knowledge and skill levels, resulting in perceptual differences in hazard management.

Gender is a common factor influencing work allocation, with tasks that are hazardous or energy consuming being typically allocated to men. A survey conducted by Council of Labor Affairs, Executive Yuan, Taiwan found that 44.7% more men than women were employed for hazardous or energy-consuming work [44]. Hazard management tasks are typically hazardous and energy consuming, and thus gender may influence perceptions concerning the frequency of handling hazards, causing the perceptions of men and women to differ.

The influence of “years of OSH experience” on the frequency of managing hazards may be similar to that discussed for “tenure at the college of employment”. Specifically, respondents with more years of service may possess higher safety awareness and hazard sensitivity, resulting in higher perceptions concerning the frequency of managing hazards than those with less years of service.

## 4. Suggestions, Directions for Future Research, and Research Limitations

### 4.1. Suggestions

Universities are one of the primary channels for fostering professionals. To avoid hazards, the researchers propose that the following suggestions serve as a reference for college managers and SPs.

1. Strengthen chemical hazard management: Study findings indicate that chemical hazards are the most common hazard type managed by college SPs, in which “organic solvents” and “other chemical substances” rank first and third of the overall hazard items. The results highlight the value of chemical hazard management. Thus, universities should strengthen chemical hazard management.
2. Identify potential hazards and conduct risk assessment and management: Based on the study results, the researchers classified hazards into physical, chemical, biological, ergonomic, and sociological and psychological hazards, allocating numerous hazard items into each type. However, potential hazards may differ depending on the situation of each college. Thus, universities should identify hazards and assess risk to determine which hazards are the most risky or volatile, thereby increasing the efficiency of hazard management.
3. Consider the influences that organizational and individual factors have on hazard management: College safety and health management is part of college organizational management. Universities that appropriately allocate labor based on potential influential factors can improve the efficiency and effectiveness of college safety and health management, thereby ensuring the safety and health of everyone on campus.
4. Improve the communication skills and interpersonal relations of SPs: Hazard management in universities frequently requires collaboration between internal and external units. Matters such as safety education, counseling and guidance, disaster rescue, and medical care cannot be completed by SPs alone. Therefore, establishing and improving the communication skills and interpersonal relations of SPs can improve the efficiency and effectiveness of hazard management.

### 4.2. Directions for Future Research

1. Cross-validation of the factor models: Only a single sample set was used for the EFA. Future studies could use another sample set for cross validation and investigate whether other sample sets exhibit similar factor patterns.
2. Employ the Delphi technique to establish hazard management content: The present study conducted a literature review, engaged in onsite observation, and employed factor analysis to establish the dimensions and items of hazard management. Future studies could employ the Delphi technique or the revised Delphi technique to collect expert opinions and more comprehensively investigate the content of hazard management.
3. Expand the research scope: The present study examined the SPs serving in Taiwan universities, and therefore the findings cannot be applied to other industries. Future studies could expand the scope of research to encompass other industries or the universities of other countries, increasing the relevance and external credibility of the findings.
4. Examine the correlation between the interpersonal relations of SPs and hazard management: Favorable interpersonal relations are essential for SPs to effectively implement hazard management. Future studies could determine which interpersonal relations directly influence the hazard management of SPs and the key correlations between interpersonal relations and hazard management.
5. Identifying the essential skills of SPs: The present study only examined the frequency of hazard management by college SPs. Future studies could determine the essential skills that individuals must possess to assume the role of an SP. In addition, having confirmed the essential skills of SPs, additional studies could determine the educational courses required to foster such skills.

### 4.3. Research Limitations

The present study investigated the hazards that college SPs may be required to manage. However, the study findings may not be generalizable to SPs in other industries. In addition, a self-reporting, paper-based questionnaire was adopted as the research tool. Although the study endeavored to maintain information confidentiality during the investigation process and guaranteed discretion to the recipients, the studied research problems were relatively controversial, which consequently created unavoidable biases because of respondents’ social expectations.

Finally, the external environment (e.g., the governmental laws and regulations) can influence employees’ behavior [45]. The data in this study are from a survey administered in 2009. The policy, laws, and regulations on occupational safety and health have been changed in Taiwan in the past seven years. For example, the Labor Safety and Health Act [18] was amended as the Occupational Safety and Health Act [46] in 2013. These changes may have an impact on the roles and functions of SPs. As such, the readers should notice that the content and frequency of hazard management dealt with by SPs may be different in the present context.

## 5. Conclusions

The present study performed an EFA on a self-developed SP HMS and extracted five factors: physical, biological, sociological and psychological, ergonomic, and chemical hazards. Subsequently, the factor-loading values, eigenvalues, and explanatory variance values of all of the factors achieved favorable standards. The results of an internal consistency analysis showed that the factors individually and collectively achieved favorable reliability. Based on these results, the HMS developed in the present study was suitable for measuring the frequency of hazard management of college SPs.

The sequential order of frequency for managing the five hazard types was chemical, physical, ergonomic, biological, and sociological and psychological hazards. Regarding individual hazard items, the 10 most frequent hazards that were identified as being managed by college SPs (in order of most to least frequent) were organic solvents, illumination, other chemical substances, machinery and equipment, fires or explosions, electricity, noise, specific chemical substances, human error, and lifting or handling. SPs within industries can now discuss plans revolving around these five areas instead of having to deal with all of the separate hazards.

One-way MANOVA results showed that the “tenure at the college of employment” and “types of certification” of college SPs achieved statistical significance and practical significance with frequency of managing hazards. Subsequently, “gender” and “years of OSH experiences” failed to achieve statistical significance but achieved practical significance with frequency of managing hazards.

## Figures and Tables

**Table 1:** The third exploratory factor analysis (EFA) results for hazard management scale.

Item	Physical	Biological	Ergonomic	Social and Psychological	Chemical
H4 Vehicles	0.76				
H18 Noise	0.70				
H3 Machinery and equipment	0.68				
H2 Falls	0.68				
H1 Electricity	0.62				
H17 Illumination	0.62				
H5 Fire and explosion	0.58				
H13 Fungi		0.85			
H12 Bacteria		0.83			
H15 Animal		0.83			
H16 Plant or insect		0.81			
H14 Virus		0.62			
H22 Working posture			0.77		
H23 Visual display			0.77		
H24 Human errors			0.70		
H21 Lifting and carrying			0.59		
H19 Vibration			0.54		
H27 Sexual harassment				0.86	
H26 Workplace violence				0.83	
H28 Alcohol or drugs				0.78	
H25 Mental workload/stress				0.65	
H11 Other chemical substances					0.79
H10 Specific chemical substances					0.79
H8 Organic solvent					0.70
Eigenvalues	4.53	4.16	3.72	3.58	3.52
Explained variance (%)	18.88	17.34	15.51	14.91	14.66
Total explained variance (%)	18.88	36.23	51.73	66.64	81.30

Note: factor loadings less than 0.50 have not been printed and variables have been sorted by loadings on each factor.

**Table 2:** Ranks of hazard management frequency.

Rank	Physical (M)	Biological (M)	Chemical (M)	Ergonomic (M)	Social and Psychological (M)	All (M)
1	Illumination (2.06)	Bacteria (1.58)	Organic solvent (2.14)	Human errors (1.75)	Mental workload/stress (1.53)	Organic solvent (2.14)
2	Machinery and equipment (1.97)	Animal (1.58)	Other chemical substances (1.97)	Lifting and carrying (1.69)	Workplace violence (1.30)	Illumination (2.06)
3	Fire and explosion (1.94)	Fungi (1.53)	Specific chemical substances (1.85)	Working posture (1.65)	Sexual harassment (1.29)	Other chemical substances (1.97)
4	Electricity (1.94)	Virus (1.51)		Visual display (1.57)	Alcohol or drugs (1.26)	Machinery and equipment (1.97)
5	Noise (1.87)	Plant or insect (1.46)		Vibration (1.51)		Fire and explosion (1.94)
6	Vehicles (1.64)					Electricity (1.94)
7	Falls (1.61)					Noise (1.87)
8						Specific chemical substances (1.85)
9						Human errors (1.75)
10						Lifting and carrying (1.69)
Total	1.86	1.53	1.99	1.64	1.34	

**Table 3:** Repeated measures one-way analysis of variance.

Variable	*N*	Mean	SD	Pairwise Comparison
Physical	144	1.86	0.93	Physical > Biological; Physical > Social and Psychological; Physical > Ergonomic; Biological > Social and Psychological; Ergonomic > Social and Psychological; Chemical > Physical; Chemical > Biological; Chemical > Social and Psychological; Chemical > Ergonomic
Biological	144	1.53	0.89	
Social and Psychological	144	1.34	0.72	
Ergonomic	144	1.64	0.91	
Chemical	144	1.99	1.06	

Notes: *N*: Sample size; SD: Standard deviation.

**Table 4:** ANOVA for job tenure difference in perceived hazard management: Physical hazards.

Source of Variation	*SS*	*df*	*MS*	*F*	*p*	*ω*^2^	1 − β	Comparison
Between	7.393	1	7.393	9.208 **	0.003	0.056	0.854	B > A
Within	109.995	137	0.803					
Total	117.388	138						

Notes: ** *p* < 0.01; A: 9 years or less; B: 10 or more years.

## Appendix

**Table A1:** Item analysis result for hazard management scale.

Item	Mean	SD	Skewness	Kurtosis	Item-Total Score Correlation
Electricity	1.94	1.18	1.17	0.38	0.84 **
Falls	1.61	1.04	1.81	2.44	0.80 **
Machinery and equipment	1.97	1.14	1.18	0.64	0.83 **
Vehicles	1.64	1.09	1.87	2.74	0.73 **
Fire and explosion	1.94	1.13	1.17	0.55	0.77 **
Ionizing radiation or non-ionizing radiation	1.58	1.01	1.99	3.45	0.77 **
Dust	1.53	0.92	2.02	3.93	0.68 **
Organic solvent	2.14	1.20	0.85	−0.28	0.78 **
Lead	1.42	0.87	2.34	5.18	0.75 **
Specific chemical substances	1.85	1.09	1.21	0.60	0.76 **
Other chemical substances	1.97	1.16	1.11	0.29	0.76 **
Bacteria	1.58	1.04	1.96	3.12	0.68 **
Fungi	1.53	1.01	2.13	3.89	0.70 **
Virus	1.51	1.04	2.21	4.07	0.67 **
Animal	1.58	1.04	1.96	3.12	0.70 **
Plant or insect	1.46	0.94	2.32	4.99	0.73 **
Illumination	2.06	1.11	1.03	0.51	0.76 **
Noise	1.87	1.04	1.23	0.97	0.74 **
Vibration	1.51	0.91	1.92	3.19	0.75 **
Cold or heat	1.54	0.96	2.00	3.66	0.76 **
Lifting and carrying	1.69	1.11	1.68	1.89	0.82 **
Working posture	1.65	1.08	1.75	2.27	0.80 **
Visual display	1.57	1.00	1.81	2.51	0.78 **
Human errors	1.75	1.06	1.59	2.08	0.74 **
Mental workload/stress	1.53	0.91	1.99	3.83	0.73 **
Workplace violence	1.30	0.77	2.99	9.17	0.75 **
Sexual harassment	1.29	0.74	3.00	9.65	0.69 **
Alcohol or drugs	1.26	0.74	3.36	11.71	0.74 **

Note: ** *p* < 0.01; SD = standard deviation.
